# The role of epithelial-mesenchymal transition and autophagy in pancreatic ductal adenocarcinoma invasion

**DOI:** 10.1038/s41419-023-06032-3

**Published:** 2023-08-07

**Authors:** Jian Yang, Ying Liu, Shi Liu

**Affiliations:** 1 Central Laboratory, The Third Affiliated Hospital, Qiqihar Medical University, Qiqihar, 161000 Heilongjiang Province P.R. China; 2 Department of Medical Oncology, The Third Affiliated Hospital, Qiqihar Medical University, Qiqihar, 161000 Heilongjiang Province P.R. China

**Keywords:** Macroautophagy, Metastasis

## Abstract

Of all pancreatic cancer (PC) cases, approximately 90% are pancreatic ductal adenocarcinoma (PDAC), which progress rapidly due to its high degree of invasiveness and high metastatic potential. Epithelial-mesenchymal transition (EMT) is a prerequisite for cancer cell invasion and spread, and it is mediated by the specific cellular behaviors and the tumor microenvironment. Autophagy has long been a target of cancer therapy, and it has been considered to play a dual and contradictory role, particularly regarding EMT-mediated PDAC invasion. This review discusses the characteristics and the biological role of EMT and autophagy from a cellular perspective, explaining invasion as a survival behavior of PDAC, with the aim of providing novel insights into targeting EMT and autophagy to overcome PDAC invasion.

## FACTS

- The purpose of PDAC invasion is seeking favorable survival.
- EMT is dynamically plastic during the progression of PDAC.
- Autophagy is a survival tool of PDAC during EMT mediated invasion.
- The role of autonomous autophagy in EMT mediated invasion of PDAC is dynamic.
- Non-autonomous autophagy provides niches for PDAC invasion.

## OPEN QUESTIONS

- How to decrease EMT plasticity to effectively inhibit PDAC invasion?
- How to overcome the dynamic role of autonomous autophagy in PDAC invasion?
- How to target non-autonomous autophagy to inhibit PDAC cell spatial plasticity?
- How to develop new effective autophagy-related drugs to overcome PDAC invasion?

## Introduction

**Fig. 1:**
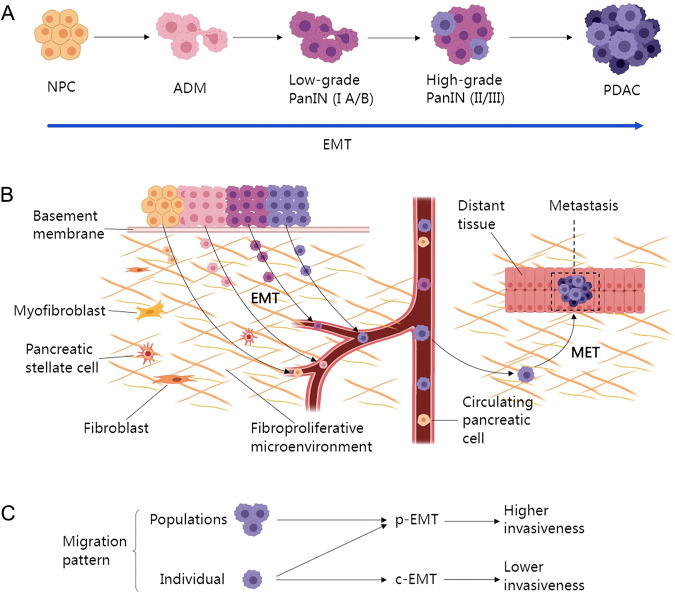
EMT and the progression of PDAC. **A** The EMT process during PDAC progression. NPC undergo ADM, progressing to low-grade PanIN (I A/B), and high-grade PanIN (II/III) finally progressing to PDAC. PDAC progression inherently results in the acquisition of EMT-associated features. **B** The inherent circulatory properties of PDAC enable it to undergo EMT at any stage in its progression. EMT is a prerequisite for PDAC invasion and diffusion, and following extravasation and colonization of a distant site, the cells undergo MET to complete the metastatic journey. **C** The migration of PDAC cells can occur via one of two EMT programs, individual and populations of cells undergo p-EMT, which results in a high invasive capacity, whilst c-EMT is achieved in an individual-cell pattern, and has a low invasive potential. ADM acinar–ductal metaplasia, c-EMT complete EMT, EMT epithelial-mesenchymal transition, MET mesenchyme-epithelial transformation, NPC normal pancreatic cell, PanIN pancreatic intraepithelial neoplasia, PDAC pancreatic ductal adenocarcinomas, p-EMT partial EMT.

Given its high invasiveness, pancreatic cancer (PC) is one of the deadliest malignancies. As of 2023, the overall 5-year survival rate of PC patients is 12%, and it remains the third leading cause of cancer-related death [1]. The median survival time of early-stage patients is 2 years following standardized surgical treatment or radiotherapy and chemotherapy, and the overall 5-year survival rate is 15–20% [2]. The 5-year survival rate of patients with advanced PC is less than 3% [3]. Approximately 90% of PC cases are classed as pancreatic ductal adenocarcinoma (PDAC) [4], and the high degree of invasiveness and high metastatic potential result in rapid progression of PDAC, with treatment having limited effects [5]. Our understanding of the occurrence and development of PDAC has gradually increased after decades of research. Acinar–ductal metaplasia (ADM), which is considered to be the primary origin of PC [6], undergoes pancreatic intraepithelial neoplasia (PanIN) I A/B, II, and III, and finally to PDAC (Fig. 1A). The most common genetic alterations observed in PDAC are *KRAS* (70–90%), *TP53* (20–76%), *CDKN2A* (49–98%), and *SMAD4* (15–50%) mutations, which promote tumorigenesis [7]. However, the most striking feature of PDAC involving progression is tumor heterogeneity.

Tumor heterogeneity best reflects the malignant potential of cancer and promotes cancer invasion and metastasis [8]. Genetic instability, differential mutational profiles, and differential gene expression patterns all affect tumor heterogeneity [9, 10]. Of course, in addition to the heterogeneity of PDAC itself, there is also spatial heterogeneity (the differential malignant potential between orthotopic and ectopic tumors), which is a result of tumor EMT.

EMT is a process by which polar epithelial cells are transformed into mesenchymal cells with a significantly enhanced ability to migrate and invade the surrounding tissues. When E-cadherin expression is reduced and cell adhesion is reduced, or functional activation of EMT allows cancer cells to shed cell-cell junctions, acquire the ability to infiltrate and invade through the basement membrane, and subsequently undergo tumor-stromal interactions to intravasate into the blood vessels [11]. When cancer cells extravasate from the blood vessels, they invade into cancer-free tissues, overcoming the interstitial or parenchymal anticancer barriers [11]. Mesenchyme-epithelial transformation (MET) subsequently occurs, and after the loss of the enhanced migratory ability possessed by mesenchymal cells, they adopt apical-basal polarization and express junction complexes to restore epithelial tissue interactions [12], thus completing the metastatic processes to distant sites [13]. These cellular processes involving EMT contribute to the understanding of the role of autophagy in the kinetics of invasion (Fig. 1B).

**Fig. 2:**
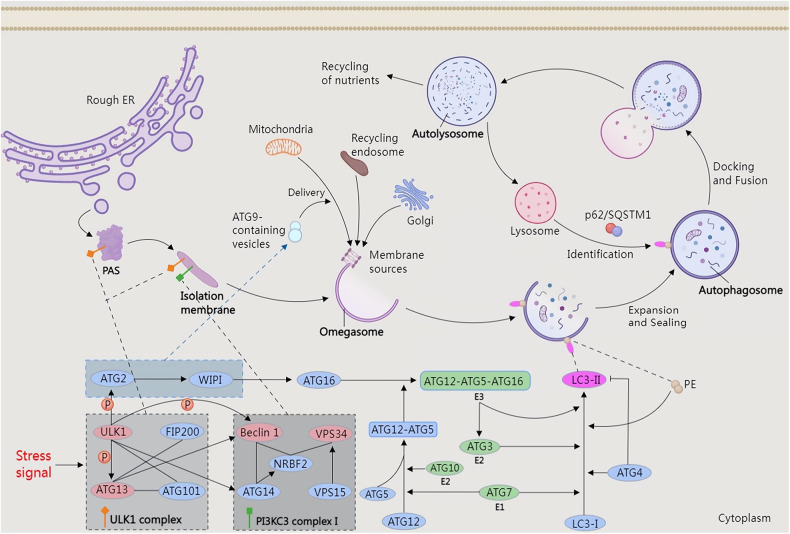
The basic process of autophagy. The activation of autophagy is typically induced by various stress signals, such as starvation, ER stress, hypoxia, protein aggregation, oxidative stress, etc. The common goal of these signals is to activate the ULK1 complex (consisting of ULK1, ATG13, FIP200, and ATG101). Autophagy is initiated at the PAS, where the ULK1 complexes are recruited. The activated PI3KC3 complex I (consisting of VPS34, Beclin 1, NFRB2, VPS15, and ATG14) then participates in the formation of the isolation membrane; ATG2 and WIPI are then activated to activate ATG9, and ATG9-containing vesicles are responsible for delivering membrane resources from the mitochondria, the Golgi, and recycling endosomes to form a characteristic ER structure called the omegasome; WIPI directly activates ATG16, then ATG12 forms an ATG12-ATG5 complex with ATG5 under the catalysis of ATG7 (E1-like enzyme) and ATG10 (E2-like enzyme), and ATG16 recruits the ATG12-ATG5 complex to form an ATG12-ATG5-ATG16 complex (E3-like enzyme); LC3-I derived from the cytoplasm binds to PE and localizes to the autophagosome membrane under the combined actions of ATG7, ATG3 (E2-like enzyme), ATG12-ATG5-ATG16 complex, and ATG4; The assembled autophagosome is extended and closed to form a mature autophagosome; ATG4 removes the remaining LC3-II after autophagosome membrane closure; this process is called the assembly and formation of autophagosomes. Under the actions of p62/SQSTM1, which has a cargo linker function, lysosomes can accurately recognize mature autophagosomes and fuse with them to form autolysosomes. Finally, the substances in the autophagylysosome are degraded, recycled, and reused. ATG autophagy-related protein, ER endoplasmic reticulum, FIP200 RB1-inducible coiled-coil protein 1, NFRB2 nuclear receptor binding factor 2, PAS phagosome assembly site, PE phosphatidylethanolamine, PI3KC3 kinase components of the class III PI3K, VPS34 consisting of vacuolar protein sorting 34, ULK1 unc-51-like kinase 1.

According to the different means of transporting cellular degradation products to lysosomes, mammalian autophagy can be divided into three types: macroautophagy, microautophagy, and molecular chaperone-mediated autophagy [14]. The lysosome-mediated autophagic process usually refers to macroautophagy [15], hereafter referred to as autophagy. Autophagy serves as a cellular defense and survival mechanism to assist in maintaining cellular homeostasis. Autophagy is involved in the maintenance of the metabolism and survival of cells during starvation and stress, and it also eliminates damaged proteins and organelles to ensure the sufficient quality and quantity of proteins and organelles [16, 17]. The basic process of autophagy involves autophagy initiation and isolation membrane formation, assembly and formation of the autophagosome, autophagosome docking and fusion with the lysosome, and autophagosome content recycling and reuse [18–22] (Fig. 2).

Autophagy has long been a target for cancer therapy, nevertheless, both activation and inhibition of autophagy can promote EMT-mediated PDAC invasion, and thus the role of autophagy is thought to be dual and paradoxical [23, 24]. Autophagy is divided into autonomous and non-autonomous autophagy, autophagy in PDAC cells is termed autonomous autophagy, and autophagy in cells outside of PDAC is termed non-autonomous autophagy [including fibroblasts and pancreatic stellate cells (PSCs) in the stroma] [25]. Autonomous autophagy maintains the EMT process in PDAC by stabilizing organelle function [26], scavenging toxic substances [26, 27], regulating metabolic homeostasis [28], buffering nutrient deprivation [29] and resisting immune recognition [30]; whereas the in situ survival of PDAC is reduced after inhibition of autonomous autophagy, accelerating PDAC to initiate EMT for favorable survival [31, 32]. Non-autonomous autophagy maintains the growth and metabolic reprogramming of PDAC after invasion by forming an ecological niche that supported EMT [33–35]. Based on a review of the literature we describe the dynamic plasticity of EMT and dynamic mechanisms of autophagy from a cell biological perspective, and provide insights into targeting EMT and autophagy to overcome PDAC invasion.

## The purpose of PDAC cell invasion

The "fertile soil" theory states that invasion is considered to be an acquired ability in the progression of cancer cells and is one of the ways in which cancer cells grow malignantly [36]. Therefore, PDAC cell invasion can be interpreted as searching for more favorable survival, and usually occurs in two ways.

One is that tissue environments can provide growth conditions for cancer cells where nutrition and living space are not restricted, and cancer cells in situ can decide whether to invade ectopically according to their own survival needs [36, 37]; the other is that the cancer cells in situ want to escape from the overload survival crisis, such as proliferation inhibition, apoptosis activation, cell metabolism disorder, attack by toxic substances (e.g. ROS and chemotherapy drugs), immune recognition, etc., the invasive behavior of cancer cells come in response to these dangerous signals. In this case, the invasion event is usually accompanied by a strong proliferative desire, apoptotic resistance and metabolic reprogramming of the cancer cells [27, 31, 38, 39]. In brief, the invasive behavior of PDAC is in fact a survival behavior. But as to when the cancer cells will invade, this depends on the response of cancer cells to the stress in the microenvironment.

## The characteristics of EMT in PDAC

EMT is considered a prerequisite for the invasion and spread of cancerous cells [40]. However, the entire PDAC biological process is accompanied by EMT features, involving in precancerous evolution of PDAC cells, tumor progression, tumor microenvironment and EMT-related molecular networks.

### The EMT program of PDAC cells

EMT-PDAC cells are PDAC cells that acquired the EMT phenotype, they initiate the EMT program immediately after detachment from epithelial cell adhesion molecules [41]. EMT-PDAC cells have circulating properties that promote the plasticity and stemness of PDAC and lay the foundation for the highly invasive nature of it [42] (Fig. 1B, C).

Rhim et al. reported in *Cell* that EMT program occurs before pancreatic tumor formation [43]. Using single-cell lineage tracing, the authors demonstrated that normal pancreatic cells (NPCs), ADM, PanIN, and PDAC cells all undergo EMT [43]. In fact, NPCs are a circulating cell type that can cross the basement membrane and enter the intravascular circulation [43]. This circulating property of NPCs persists throughout the progression of pancreatic cells to PDAC and maintains the mesenchymal phenotype [43]. When pancreatic tumor cells enter a circulating state, they acquire functions similar to those of pancreatic cancer stem cells, namely, enhanced tumor initiation and differentiation capacity after transplantation into a permissive host [43]. Moreover, EMT-mediated invasive behavior occurs before tumor formation and is difficult to detect histologically [43]. These findings explain how PDACs can metastasize at an early stage of development.

The EMT program affects cellular plasticity. Zheng et al. reported in *Nature* that the EMT program of PDAC enhances tumor cell survival following gemcitabine treatment and protects tumor cells from survival crises [27]. However, the inhibition of EMT program at transcriptional level did not alter PDAC progression, local invasion, systemic diffusion rates, and metastasis [27]. Indeed, two distinct EMT programs mediate different modes of PDAC invasion, Partial EMT (p-EMT): a program that does not undergo complete conversion to a mesenchymal phenotype, that is, it can continue to express epithelial genes after undergoing EMT, increasing the malignant invasive potential of PDAC by regulating the migration patterns of individual cells and cell clusters [44]; and Complete EMT: (c-EMT): a mesenchymal program in which cells almost completely lose their epithelial phenotype, and typically regulate individual cell migration patterns, resulting in a relatively low invasive potential of PDAC [44]. The results indicated that most of PDAC cells lose their epithelial phenotype via the p-EMT model (transcription-dominated program), whilst only a minority of tumor cells lose their epithelial phenotype via the c-EMT model (protein relocation-dependent), and that neither model affects the ability of cancer cells to undergo MET [44]. These findings suggest different modes of invasion associated with different EMT programs, providing an explanation for why cellular plasticity (epithelial to mesenchymal cell transition) drives invasion.

### PDAC invasion in the tumor microenvironment

**Fig. 3:**
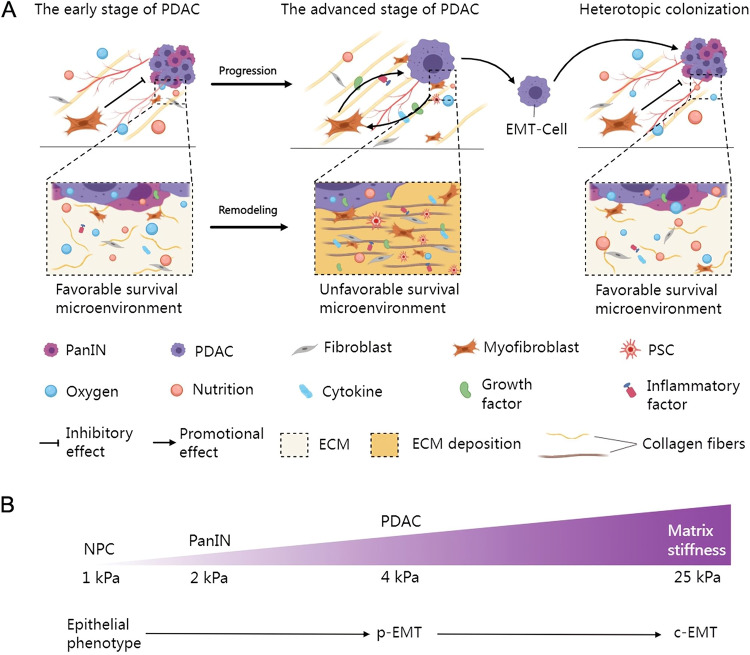
EMT characteristics in the tumor microenvironment of PDAC. **A** In the early stage of PDAC, mesenchymal cells inhibit tumor growth, meanwhile, oxygen, nutrients and blood supply are sufficient for PDAC progression in the tumor microenvironment. However, by the time PDAC has progressed to advanced stage, the microenvironment has been remodeled, including collagen fibers alignment and thickening, increased stromal cells activation and number, and ECM deposition. Activated stromal cells and cancer cells promote each other by releasing inflammatory factors, cytokines and growth factors. The remodeled microenvironment results in inadequate blood, oxygen and nutrient supply to the tumor. Therefore, PDAC is prone to search a favorable survival, that is EMT and invasion process. **B** The effect of EMT on matrix stiffness during PDAC progression, data from Rice et al. [61]. c-EMT complete EMT, EMT epithelial-mesenchymal transition, ECM extracellular matrix, NPC normal pancreatic cell, PDAC pancreatic ductal adenocarcinomas, p-EMT partial EMT.

PDAC cells and tumor microenvironment are interacting during PDAC progression. The unfavorable survival environment such as hypoxia, nutrient deprivation, and inflammation promotes EMT mediated invasion of PDAC, in turn, the invasion of PDAC reshapes the tumor microenvironment (Fig. 3).

PDAC progression affects microenvironment remodeling. PDAC tissues have to overcome severe hypoxia and extremely low nutrient availability [45]. From PanIN to PDAC, the stroma resists tumor progression driven by genetic mutations and inhibits tumor proliferation [46]. From established PDAC to advanced stages of development, specific hypovascular perfusion [47] within the tumor and the surrounding stroma severely limits oxygen utilization and nutrient uptake of PDAC [45]. The surrounding stroma cells are activated such that the stroma-derived signals, cytokines and growth factors outweigh PDAC-derived ones, and EMT niche formation drives tumor invasion to reshape the microenvironment [48]. Subsequently, some of the changes involved in the stroma include angiogenesis in the interstitium to meet the oxygen and nutrient demands of the tumor [49, 50], secretion and transport of alanine to support tumor metabolism [33], interstitial fibroproliferative barrier formation to resist immune cell attack [45], and tumor-stroma inter-transduction signaling activation to promote tumor growth [51], together form a microenvironment promoting survival and tumor invasion.

Tumor-stroma interactions provide the niche for EMT in PDAC. PDAC is characterized by a fibroproliferative microenvironment with fibroblasts as the primary cell type [45]. Cancer-associated fibroblasts (CAFs) are a heterogeneous population of cells that interact with tumor cells [52, 53]. PC cells activate fibroblasts by secreting growth factors, cytokines, and exosomes; in-turn, activated CAFs produce extracellular matrix (ECM), growth factors, and inflammatory factors to promote stemness, invasion, and metastasis of tumors [54]. Of note, tumor-stromal interactions are direct. Myofibroblasts are a type CAFs, present at the tumor-stroma interface, and they act in an anchorage-independent manner to promote tumor cell self-renewal and proliferation, providing a niche for adjacent tumor cells to achieve EMT [8].

An imbalance (increase or decrease) in the number of activated mesenchymal cells increases PDAC invasion. Unlike tumor cells, stromal cells have not undergone transformation, and thus more attention has been paid to the study of targeting stroma in PDAC invasion kinetics [55]. PSCs are primary stromal cells capable of transforming from a quiescent state into hyperactivated myofibroblasts, which form the stromal microenvironment together with the ECM. PSCs not only promote tumor-stromal interactions [56], but also secrete growth factors and cytokines to promote pancreatic cancer proliferation and invasion [57]. The total percentage of PSCs in the stromal cell population increases, leading to ECM contraction, resulting in progressive accumulation of surrounding fibrous connective tissue, activation of the EMT phenotype, and subsequent remodeling of the microenvironment to promote tumor invasion and survival [58]. However, another study suggested the use of a cautious strategy when targeting the interstitium because myofibroblast depletion can inhibit angiogenesis, reduce overall immune infiltration, increase tumor hypoxia and necrosis, furthermore the unfavorable survival microenvironment can cause cancer cells EMT and acquiring stemness [59]. This suggests that the survival crisis caused by the decreased numbers of activated stromal cells increases the invasive behavior of PDAC.

Changes in interstitial stiffness promote EMT in PDAC. Tumors are often found to be firm during surgical dissection. Indeed, the tumor-induced increase in matrix stiffness promotes invasion. Interstitial fibrosis and ECM deposition result in changes in stromal cell mechanics [60], including increased tissue tension, collagen fiber alignment and thickening, furthermore, promote PDAC invasion and metastasis by reducing spatial impedance. These suggest that EMT is spatially plastic instead of a simple epithelial-mesenchymal qualitative binary transformation typically proposed in many studies [61]. However, a decrease in matrix stiffness promotes PDAC invasion likewise. Myofibroblast depletion reduces the content of type I collagen fibers, leading to a decrease in the matrix stiffness and elastic modulus [59]. In this context, the expression of Vimentin (mesenchymal marker) is reduced, nevertheless, the EMT program is initiated to promote tumor cell invasion [59]. That is, the increased matrix stiffness promotes EMT, but decreased stiffness does not impede the EMT-mediated invasive program in PDAC.

An inflammatory microenvironment promotes progression and EMT in PDAC. The interstitium is infiltrated by a large number of inflammatory cells and immune cells (macrophages, neutrophils, dendritic cells, myeloid-derived suppressor cells, B cells, and T cells) in addition to the above-mentioned fibrous and collagenous components [62]. They are recruited to secrete chemokines and cytokines that promote tumor growth in response to signals from *KRAS* mutations in tumors [62, 63]. Inflammation promotes tumor formation and accelerates the progression of PanIN to PDAC by inhibiting *KRAS*-induced senescence [64, 65]. Certain inflammatory factors mediated by nuclear factor-κB (NF-κB) or signal transducer and activator of transcription 3 (STAT3), such as interleukin 1 (IL-1), tumor necrosis factor α (TNFα), and IL-6 promote EMT [62, 66], and treatment of inflammation with glucocorticoids can inhibit EMT and reduce PDAC invasion and metastasis [43].

### EMT-related transcription factors and pathways involving in the invasion kinetics of PDAC

The EMT program is often activated by a number of EMT-related transcription factors (EMT-TFs), mainly including snail family transcriptional repressor 2 (*Slug*, also known as *Snai2)*, twist family BHLH transcription factor 1 (*Twist)*, snail family transcriptional repressor 1 (*Snail*, also known as *Snai1)*, and zinc finger E-Box binding homeobox 1/2 (*Zeb1/2*). Greco et al. recently described EMT-TFs, and related signaling pathways mediated by factors such as transforming growth factor-β (TGF-β), epidermal growth factor (EGF), fibroblast growth factor (FGF) and hepatocyte growth factor (HGF), Wnt/β-catenin, hedgehog, notch, and TNF [67, 68].

EMT procedures histologically reveal invasive behavior in PDAC. Hotz et al. observed higher expression levels of *Snail* than *Slug* in human PC tissues, and both two were not expressed in normal tissues. In addition, the lower the degree of PC cell differentiation, the higher the level of *Snail* expression and the concomitant loss of E-cadherin. This is consistent with the fact that the higher the expression levels of EMT-TFs in PDAC the more invasive it is [69]. *Twist* was neither expressed nor methylated in PC tissues or cell lines, but hypoxia was able to significantly upregulate its expression to trigger invasive events [69]. This explains histologically that an unfavorable survival environment (hypoxia) favors the initiation of the EMT program in PDAC. Furthermore, the expression levels of EMT-TFs correlated with tumor site, *Snail* mainly expressed in ductal cells at the center of the tumor, while *Slug* was mainly found in the infiltrating front of the tumor [69]. This suggests that Snail expression is involved in the induction of invasion, while Slug is involved in the maintenance of the invasive phenotype of PDAC cells [69]. Krebs et al. used a KPC mouse model (Pdx1-cre-mediated activation of mutant *Kras* and *p53*) to show that *Zeb1* is expressed in a fraction of PDAC cells and precancerous lesion cells [42]. The authors also demonstrated that Zeb1 expression promoted post-invasive events in PDAC, that is, enhanced stemness, tumourigenicity and metastatic capacity of cancer cells [42]. Collectively, EMT-TFs play different roles in the different steps of the PDAC invasion process.

Deficiency of EMT-TFs affects EMT plasticity in PDAC invasion. Deletion of *Zeb1* allows PDAC cells to transform from a mesenchymal phenotype to an epithelial cell phenotype (reduced EMT plasticity) and is less responsive to TGF-β-regulated signaling pathways [42]. However, loss of *Zeb1* did not prevent PDAC invasive events, as *Zeb1* deletion (epithelial phenotype predominant) also significantly increased local invasion and liver metastasis [70]. Zheng et al. deleted a single *Twist1* or *Snai1* gene did not affect PDAC tumorigenicity, stemness, local invasion and distant colonization ability [27]. This indicates, to some extent, that individual *Twist1* or *Snai1* has a weaker effect on EMT plasticity than *Zeb1*. Carstens et al. suggested that simultaneous gene deletion of *Twist1* and *Snai1* suppressed p-EMT and mesenchymal phenotype by affecting the entire EMT transcriptional profile, maintaining the epithelial phenotype into a steady state, just like *Zeb1* deletion [70]. However, this stable epithelial phenotype of PDAC cells can still accomplish invasive and metastatic behavior in a population migration manner [70]. Taken together, these findings suggest that the EMT program is highly plastic, i.e., epithelial phenotype, p-EMT, and mesenchymal phenotype exist as an interchangeable continuum during PDAC invasion. The regulation of EMT plasticity by individual or multiple transcription factors is heterogeneous.

**Fig. 4:**
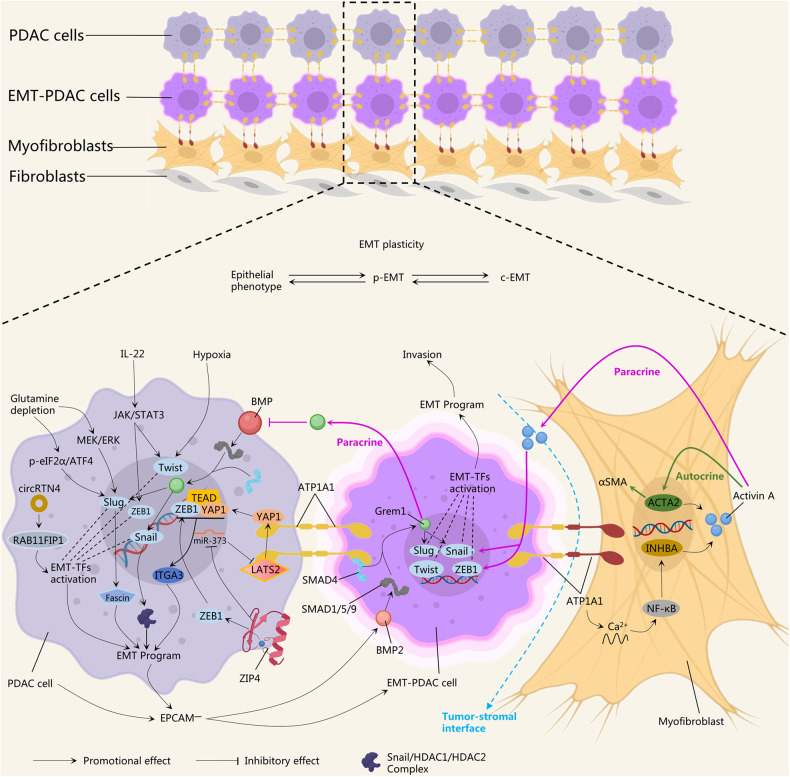
EMT-TFs and relative pathways in PDAC invasion kinetics. EMT plasticity is maintained by continuous interchangeability between the epithelial phenotype, p-EMT, and c-EMT. PDAC cells lose EPCAM (EPCAM^—^) when initiating the EMT program, at which point the cancer cells transform into EMT-PDAC cells. In PDAC cells, the EMT program is usually activated by EMT-TFs (single or multiple expression). In addition, the EMT program can also be activated by the downstream pathways of EMT-TFs, including Fascin, which is regulated by Slug; the Snail/HDAC1/HDAC2 complex, which is formed by Snail; ITGA3, regulated by the binding of ZEB1 to YAP1/TEAD. For EMT-TFs, the upstream signals are activated by glutamine depletion, hypoxia, inflammation (IL-22), circRTN4 and ZIP4. Glutamine depletion activate Slug by triggering MEK/ERK and p-eIF2α/ATF4; hypoxia is able to upregulate Twist; IL-22 activate Twist and ZEB1 by inducing the JAK/STAT3 pathway; ZIP4 promotes ZEB1 binding to YAP1/TEAD to form the upstream activator of ITGA3 via two routes, one is activating ZEB1 and the other is indirectly activating YAP1 by inhibiting miR-373, the inhibitor of LATS2; circRTN4 activates EMT-TFs (Slug, Twist, ZEB1, Snail) by stabilizing RAB11FIP1. In EMT-PDAC cells, the initiation of EMT program activates BMP2 signaling, which specifically upregulate Grem1 through upregulation of SMAD1/5/9 under the involvement of SMAD4. Grem1 promoted EMT-PDAC cell invasion by upregulating downstream Slug and Snail expression, meanwhile, it inhibites BMP signaling in neighboring PDAC cells to maintain their epithelial phenotype via paracrine. Direct contact between EMT-PDAC cells and myofibroblasts results in the reorganization of the plasma membrane protein ATP1A1 at the tumor-mesenchymal interface, which activates the NF-κB signaling by triggering Ca^2+^ oscillations in myofibroblasts, NF-κB signal stimulates the secretion of activin A by INHBA. Activin A upregulates Snail and ZEB1 in adjacent EMT-PDAC cells via paracrine secretion. Activin A itself also promotes activin A production and myofibroblast fibrosis (αSMA upregulation) by stimulating ACTA2 via an autocrine pattern. ACTA2 actin alpha 2, ATF4, activating transcription factor 4, ATP1A1 ATPase Na^+^/K^+^ transporting subunit alpha 1, BMP2, bone morphogenetic protein, c-EMT complete EMT, EMT epithelial-mesenchymal transition, EPCAM epithelial cell adhesion molecule, EMT-TFs EMT-related transcription factors, HDAC histone deacetylase, IL interleukin, INHBA inhibin subunit beta A, ITGA3 integrin α3, JAK janus kinase, LATS2 large tumor suppressor kinase 2, MAPK mitogen-activated protein kinase, MEK MAPK kinase, PDAC pancreatic ductal adenocarcinomas, p-EMT partial EMT, NF-κB nuclear factor kappa B, Slug snail family transcriptional repressor 2, Snail snail family transcriptional repressor 1, PDAC pancreatic ductal adenocarcinomas, p-eIF2α phosphorylated eukaryotic initiation factor 2α, PSC pancreatic stellate cell, RAB11FIP1 RAB11 family interacting protein 1, STAT3, signal transducer and activator of transcription 3, TFEB transcription factor EB, Twist twist family BHLH transcription factor 1, YAP1 yes-associated protein 1, Zeb zinc finger E-Box binding homeobox, ZIP4 solute carrier family 39 member 4.

EMT-TFs-related pathways promote PDAC invasion by regulating EMT plasticity. Von et al. showed that EMT is dependent on histone deacetylase (HDAC) activity and that PC cells involving EMT silenced e-calmodulin through upregulation of a complex containing *Snail*, HDAC1 and HDAC2 [71]. Moreover, *Slug* was able to activate its co-expression factor Fascin (an actin-binding protein), which promotes hepar, diaphragm, mesentery, and ascites metastasis of PDAC [72]. The invasion process of PDAC is often accompanied by nutrient deprivation, which makes the invasion behavior more motivated. Upon glutamine depletion, two signaling pathways MAPK kinase (MEK)/extracellular signal-regulated kinase (ERK) and phosphorylated eukaryotic initiation factor 2α (p-eIF2α)/activating transcription factor 4 (ATF4) were activated, then Slug was activated by them to facilitate the EMT and metastasis of PDAC [73]. IL-22 upregulated ZEB1 and Twist1 expression through activation of the janus kinase (JAK)/STAT3 pathway, maintaining EMT plasticity during PDAC invasion [74]. *circRTN4* (a circular RNA derived from exons 4 and 5 of Reticulon 4 mRNA) upregulates Zeb1, Snai1, Twist, and Slug by stabilizing RAB11 family interacting protein 1 (RAB11FIP1) to promote invasion, migration, liver metastasis and apoptosis resistance of PDAC cells [38]. Solute carrier family 39 member 4 (ZIP4) activates integrin α3 (ITGA3) by regulating the co-activation of ZEB1 and yes-associated protein 1 (YAP1) to promote EMT, tumor colonisation and organogenesis in vitro and vivo models of PC [75]. Recently, critical mechanisms of EMT plasticity for maintaining celluar heterogeneity of PDAC have been revealed. Lan et al. reported in Nature that the deletion of a key regulatory “switch” that maintains epithelial fate, Gremlin 1 (*GREM1)*, results in the conversion of PDAC from an epithelial phenotype to a mesenchymal phenotype in just a few days; GREM1 secreted by EMT-PDAC cells inhibits EMT by suppressing the BMP signaling pathway in neighboring epithelial PDAC cells, GREM1 and BMP2 form a negative feedback loop between these two cells, regulating PDAC invasion in a sustained paracrine signaling pattern [41]. Moreover, direct contact between EMT-PDAC cells and myofibroblasts stimulates the secretion of activin A. Activin A promotes the initiation of the EMT program (upregulating the expression of Snail and ZEB1) and the differentiation of myofibroblasts in neighboring cancer cells in a paracrine pattern; on the other hand, it promotes the secretion of activin A by actin alpha 2 (ACTA2) in myofibroblasts in an autocrine pattern [8]. Collectively, the hierarchical EMT plasticity regulating molecular networks participate together in the maintenance of PDAC spatial heterogeneity (Fig. 4).

In summary, both in terms of PDAC itself and the tumor microenvironment, EMT is extremely likely to occur. EMT is dynamically plastic in the progression of PDAC [76], and the invasion mediated by it is a prominent means in maintaining tumor spatial heterogeneity [42, 48, 50, 58, 77, 78].

## The role of autonomous autophagy in PDAC invasion kinetics

Histologically, aggressive PDACs often experience a survival crisis such as tumor necrosis [79], which is determined by the PDAC-specific survival environment (hypovascularity and fibrosis) [45, 63]. When tumor survival is threatened, ectopic aggressive behaviors manifest with the aim of improving survival [37]. Autonomous autophagy is one such mechanism PDAC tissues utilize, assisting tumor cells in absorbing nutrients from the blood and removing excess and faulty cellular components to improve survival [80]. Currently, the controversy over the role of autonomous autophagy on PDAC invasion is focused on the both high and low levels of autonomous autophagy can promote EMT. Next, we elaborate the dynamic mechanism of autonomous autophagy in PDAC invasion kinetics in terms of the establishment of autonomous autophagy system in cancer cells, activation and inhibition of autonomous autophagy.

### Establishment of autonomous autophagy homeostasis in PDAC

Autonomous autophagy homeostasis is gradually established during the tumor progression and matures when the tumor progresses to PDAC.

Autonomous autophagy activation is present at each stage of PDAC progression, where it plays different roles [31]. During the early stages of PDAC progression (from ADM to PanIN-II), autonomous autophagy activation inhibits tumor initiation and progression; while in the later stages (from PanIN III to invasive PDAC), it promotes tumor growth and invasion [26, 31, 81, 82]. As a tumor suppressor gene, *p53* has multiple functions, including inhibiting cell cycle progression, promoting apoptosis, repairing DNA, and inhibiting angiogenesis [83]. The role of autonomous autophagy in the early stages of PDAC progression is unstable and susceptible to the actions of *p53*. In the absence of *p53*, basal autonomous autophagy in tumors inhibits tumor progression resulting in its arrest at PanIN-II [81]. This suggests that autonomous autophagy is not utilized by tumor cells in this process. When *p53* is activated, autonomous autophagy accelerates tumor progression from PanIN to PDAC [81, 82]. This suggests that the autonomous autophagy system gradually becomes part of the mechanisms by which the tumor maintains homeostasis at this stage, helping tumors resist tumor suppressor attacks [39]. For established PDACs with mature survival mechanisms (proliferation, metabolism, invasion, and metastasis) [39], maturation of the autonomous autophagy system promotes PDAC progression-free from the tumor suppressor functions of *p53* [82].

### Autonomous autophagy as a survival tool exploited by invasive PDACs

Based on the fertile soil theory [36, 37], the increase in the utilization of autonomous autophagy by PDAC cells as a response to survival crises (proliferation inhibition, apoptotic threat, toxic attack, and metabolic disturbance) results in the acquisition of invasive behaviors.

Autonomous autophagy supports survival in situ prior to invasion. In PDAC, autonomous autophagy levels are usually elevated [25, 26, 29, 30, 79, 84]. Autonomous autophagy allows cancer cell proliferation by scavenging reactive oxygen species, alleviating DNA damage, and maintaining cellular metabolism [26]. In addition, autonomous autophagy reduces the ability to present antigens by downregulating MHC-I expression and improving the fitness of cancer cells; thereby evading immune recognition by CD8^+^ cytotoxic T lymphocytes [30]. The levels of metabolism can reflect the survival potential of the tumor [85]. Autonomous autophagy maintains the normal degradation of mitochondria in cancer cells and stabilizes the activity of the tricarboxylic acid cycle and oxygen consumption to protect mitochondrial function, thereby assisting PDAC biosynthesis and energy homeostasis [28]. Moreover, MiT/TFE proteins [including melanocyte inducing transcription factor (MITF), transcription factor binding to IGHM enhancer 3 (TFE3), and transcription factor EB (TFEB)] [86], the master molecules of metabolic reprogramming, enable lysosome-autophagy system-dependent activation to buffer amino acid levels in cancer cells under conditions of starvation, thereby promoting PDAC growth [29]. Furthermore, autonomous autophagy protects PDAC from loss of survival pathways. ERK protein is part of the mitogen-activated protein kinase (MAPK) family, and the MAPK/ERK pathway is activated at the end of the RAF → MEK → ERK kinase pathway, supporting a variety of survival-related cellular behaviors [87]. The RAF → MEK → ERK pathway plays an important role in PDAC growth and progression [87]. However, inhibition of this pathway does not result in any clinical benefits for PDAC patients [88], as autonomous autophagy is activated to protect PDAC from the cytotoxic effects of inhibition of this pathway [89]. Based on autonomous autophagy as a survival protection mechanism, simultaneous inhibition of autonomous autophagy and this pathway proved to be an effective strategy for the treatment of PDAC [90]. Thus, Autonomous autophagy supports the growth, metabolism and immune escape of PDAC in situ.

Autonomous autophagy helps PDAC cells survive during invasion. TGF-β is an important regulator of cancer growth and is known for its dual roles in the survival of cancerous cells [91, 92]. During the progression from PanIN-I to PanIN-II, the TGF-β pathway prevents tumor growth; whereas, during the progression from PanIN-III to invasive PDAC, it promotes tumor growth [93, 94]. SMAD4 proteins are important co-transcription factors (mediators of the TGF-β pathway) and they play an important role in maintaining cell growth, differentiation, and tissue homeostasis [91]. In a recent study, the effect of autonomous autophagy on PDAC progression was dependent on SMAD4 status [95]. In SMAD4-positive PDAC cells, autonomous autophagy (activated by TGF-β) promotes proliferation and inhibits migration by reducing nuclear translocation of SMAD4; in SMAD4-negative PDAC cells, autonomous autophagy (activated by TGF-β) modulates MAPK/ERK activation to inhibit proliferation and promote migration [95]. This shows that PDAC cells exhibit invasive behaviors to seek favorable survival conditions, and autonomous autophagy is an auxiliary tool for PDAC survival. When the growth-promoting signals TGF-β and SMAD4 are sensed by PDAC cells, the survival conditions at this time are favorable for tumor cells, and autonomous autophagy assists tumor cell growth instead of invision; when SMAD4 deletion results in unfavorable survival conditions for tumor cells, TGF-β induces autonomous autophagy to activate another compensatory survival pathway, the MAPK/ERK pathway, and at the same time autonomous autophagy activates EMT to increase the invasive behaviors to avoid survival crisis. Indeed, SMAD4 depletion induces increased mitochondrial fragmentation and reduced oxidative metabolism in PDAC leading to mitochondrial dysfunction [96], and the adverse outcome of mitochondrial dysfunction is apoptosis [96–98]. Autonomous autophagy maintains a relatively stable mitochondrial quantity and quality by removing dysfunctional mitochondria in tumor cells, thereby maintaining sufficient energy for invasion by cancer cells to evade potential apoptotic threats [96, 99]. Thus, irrespective of the mechanism, autonomous autophagy is a survival tool utilized by invasive PDAC.

### Autonomous autophagy defects drive EMT-mediated PDAC invasion

As mentioned above, autonomous autophagy is a survival tool utilized by PDAC, thus autonomous autophagy deficiency represents a defect in the PDAC tissue’s ability to cope with a state of survival crisis, which thus drives tumors to invade new tissues to acquire new initiating abilities and undergo cell reprogramming.

Inhibition of autonomous autophagy was recently shown to promote EMT-mediated PDAC invasion [32]. In *RAS*-mutated PDACs, inhibition of autonomous autophagy specifically assisted in tumor cell detachment from neighboring cells and in the invasion of new sites [32]. Mechanistically, autonomous autophagy activates the SQSTM1/RELA pathway (NF-κB canonical pathway) [100, 101] and cooperates with *KRAS* to promote EMT [32]. The NF-κB protein is a key regulator of immune and inflammatory responses, inhibiting apoptosis, and promoting cell proliferation, invasion, metastasis, and angiogenesis [102]. NF-κB-mediated inflammation is closely related to a poor prognosis in PDAC [103–107], and inflammatory factors activate NF-κB in a positive feedback loop [106]. Meanwhile, the accumulation of SQSTM1 can aggravate pancreatic inflammation [108]. Although the authors did not study inflammation-related cellular changes, they showed that autonomous autophagy deficiency attenuated PDAC resistance to inflammation-related signals, thereby accelerating the EMT program. This is similar to the previous findings treatment of inflammation with glucocorticoids can inhibit EMT and reduce PDAC invasion and metastasis [43]. Additionally, another study showed the cellular behaviors of autonomous autophagy in this context [31]. The authors constructed an *ATG5* loss of heterozygosity by using cell line and animal model and performed comprehensive transcriptomic and metabolomic analyses. Autonomous autophagy inhibition (heterozygous deletion rather than homozygous deletion) enhanced cancer cell survival responses, including enhanced tumor cell functions related to metabolism, immunity, development, and vesicle trafficking/homeostasis; enhanced pre-EMT steps i.e. decreased cancer cell adhesion; increased cancer cell survival crisis, decreased cell cycle progression, impaired mitochondrial function, increased inflammation; increased intracellular Ca^2+^ response, and increased extracellular lysosomal cathepsin activity associated with metastasis [31]. It is worth noting here that autonomous autophagy inhibition does not prevent PDAC occurrence and invasion [109], but only increases DNA damage and apoptosis in tumors that have already formed [82]. Taken together, these findings suggest that autonomous autophagy inhibition deprives PDAC of necessary survival tools, forcing tumors to increase survival responses and initiate invasion in an effort to increase its survival probability.

**Fig. 5:**
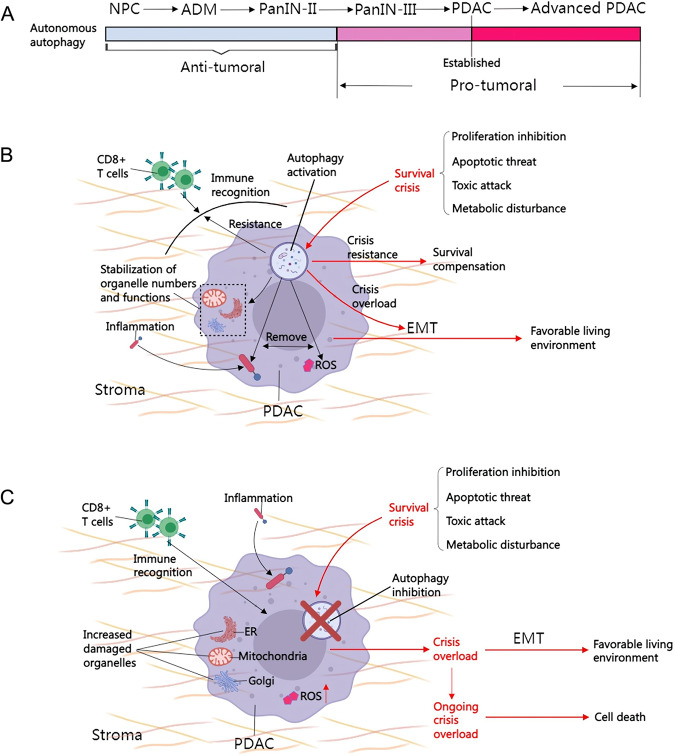
The role of autonomous autophagy in PDAC invasion kinetics. **A** Establishment of the autophagy system utilized by PDAC. From NPC to PanIN-II, autonomous autophagy suppresses tumors (shown in gray). Autophagy promotes tumor progression from PanIN-III to advanced PDAC (shown in pink and red). In PDAC stage, tumors establish a mature autonomous autophagy homeostasis system. **B** Autonomous autophagy is utilized by PDAC tissues as a means of tumor survival. Autonomous autophagy maintains tumor survival through immune escape, clearance of inflammation and ROS, and stabilization of organelle numbers and function. When tumors face a survival crisis (proliferation inhibition, apoptotic threat, toxic attack, and metabolic disturbance), autophagy promotes compensatory mechanisms to maintain tumor survival. When the survival crisis mechanisms are overloaded, autonomous autophagy assists tumor invasion for favorable survival. **C** When autonomic autophagy is inhibited, cancer cell will suffer from immune recognition, stimulation of inflammation and ROS, as well as increased organelle damage; By now the survival crisis is prone to overloading, and tumor is extremely likely to invade for favorable survival. If the survival crisis overloading continues, it will result in cancer cell death. Only autophagosomes are used in the figure to represent the autophagic process. The red arrows in the figure represent cell behaviors associated with survival crisis. EMT epithelial-mesenchymal transition, ER endoplasmic reticulum, NPC normal pancreatic cell, PanIN pancreatic intraepithelial neoplasia, PDAC pancreatic ductal adenocarcinomas, ROS reactive oxygen species.

In summary, PDAC utilizes the established autonomous autophagy system as a survival tool to maintain its own growth. The low vascular supply of PDAC itself will cause the tumor to face a survival crisis during its growth [79], and at this time, autonomous autophagy will assist PDAC in completing the invasion process. When this survival tool is lost, PDAC will break away from the disadvantaged position and invade new settlements for cellular reprogramming. Therefore, the role of autonomous autophagy in EMT-mediated PDAC invasion kinetics is dynamic, that is, either activation or inhibition of autonomous autophagy promotes PDAC invasion (Fig. 5).

## Non-autonomous autophagy provides niches for invasive PDACs

In contrast to the dynamic role of autonomous autophagy, the role of non-autonomous autophagy is relatively stable in the behavior of PDAC invasion, that is, it supports PDAC invasion from multiple aspects.

**Fig. 6:**
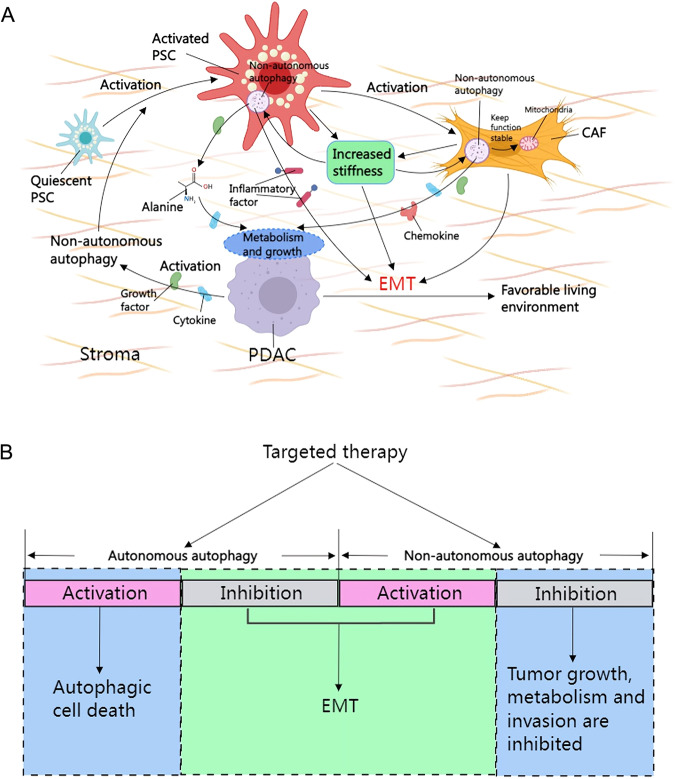
The mechanism of non-autonomous autophagy and current status of targeted autophagy therapy in EMT-mediated invasion of PDAC. **A** Signals released by PDAC induce non-autonomous autophagy activation in the stroma, converting quiescent PSC to activated PSC, further activating CAF. Non-autonomous autophagy-dependent secretion of alanine, growth factors, chemokines, and cytokines, forms a niche that supports adjacent tumor growth and metabolism. Furthermore, non-autonomous autophagy activation supports EMT-mediated invasion by promoting inflammatory factors secretion and tumor stiffness. Only autophagosomes are used in the figure to represent the autophagic process. **B** Both autonomous autophagy inhibition and non-autonomous autophagy activation promote EMT (the green area), and this underlies the lack of success of current autophagy inhibitors in the clinic. Activation and inhibition of autophagy are indicated in pink and gray, respectively. Autophagic cell death and specifically targeted inhibition of non-autonomous autophagy may be effective strategies for the treatment of PDAC (blue areas). CAF cancer-associated fibroblast, EMT epithelial-mesenchymal transition, PDAC pancreatic ductal adenocarcinomas, PSC pancreatic stellate cell.

The level of non-autonomous autophagy in the peripheral tissue of invasive pancreatic cancer is higher than that in the tumor, providing nutritional support for PDAC invasion [79]. When tumor cells release inducing signals, non-autonomous autophagy in the stroma is activated to convert quiescent PSCs into hyperactivated CAFs [34, 58, 110]. Mechanistically, non-autonomous autophagy maintains proline biosynthesis within the mitochondrial NADPH pool of CAFs for sustained activation of CAFs [34]. Stromal alterations brought about by non-autonomous autophagy activation form a niche that supports PDAC invasion and migration, promoting tumor-stroma interactions by increasing the production of IL-6 and ECM proteins [35]. However, the formation of invasion imposes extra metabolic demands that PDAC must meet for it [39]. Collagen deposition in the stroma increases its stiffness [111, 112], and forms a niche supporting PDAC metabolism by secreting growth factors, cytokines, and chemokines [113–115]. Among the 200 associated metabolites, PDAC primarily takes up the non-essential amino acids alanine and aspartate [33]. Alanine is secreted by PSCs in an non-autonomous autophagy-activation-dependent manner and is taken up by PDAC tissues in quantities 5 times greater than that of aspartate [33]. Therefore, alanine, as a major nutrient, supports the non-autonomous autophagy-dependent metabolic requirement of PDAC. Additionally, activated interstitial components induce non-autonomous autophagy activation through increased interstitial stiffness, thereby supporting adjacent cancer cell growth [116]. This tumor-mediated tumor-stroma autophagy-dependent signaling crosstalk promotes PDAC invasion and survival in a positive feedback manner. It is worth noting here that inhibition of non-autonomous autophagy does not affect the proliferative behavior of PDAC and the apoptotic behavior of CAF, but hinders the invasive behavior of PDAC [34, 35]. Taken together, non-autonomous autophagy provides more favorable survival conditions for invasive PDAC (Fig. 6A).

## Efficacy of targeting autophagy on PDAC invasion in clinical trials

As the widely recognized need for autophagy in invasive PDAC, current clinical trials focus on the use of autophagy inhibitors combined with chemotherapy for the treatment of PDAC. Based on mentioned above, the role of autophagy in PDAC invasion kinetics is dynamic, and autonomous autophagy inhibition accelerates PDAC invasion. Therefore, the clinical efficacy of autophagy inhibitors in patients with invasive PDAC may be poor.

Prospective clinical trials completed in recent years on autophagy inhibition for the treatment of PDAC are listed in Table 1. Chloroquine (CQ) and Hydroxychloroquine (HCQ) are frequently used autophagy inhibitors in current clinical trials. HCQ alone did not significantly benefit survival outcomes in patients with metastatic PDAC, as HCQ alone did not inhibit autophagy sufficiently in PC patients, or the inhibitory effect of HCQ was offset by abnormal activation of autophagy (activated by prior chemotherapy) [117]. This suggests that the effect of autophagy on metastatic PDAC is nonspecific, and HCQ alone is not suitable for the treatment of metastatic pancreatic cancer. The use of autophagy inhibitors in combination with chemotherapeutics was well tolerated [118–120]. However, autophagy inhibition combined with chemotherapy for metastatic PDAC did not significantly improve patient survival outcomes and resulted in enhanced tumor pathological responses [121, 122]. The Phase I clinical trial (CQ+gemcitabine) results reported by Samaras et al. indicated good clinical efficacy, but the data generated by the small number of subjects were unreliable [120]. These lines of evidence of well-tolerated and partially effective autophagy inhibition treatment may suggest that autophagy plays a role in a locally advanced setting but autophagy inhibition treatment contributes little to survival in patients with PDAC.

**Table 1:** Completed clinical trials of autophagy for PDAC.

Year	Study types	Number of patients	PDAC types	Treatment	Survival outcomes	Efficacy-related conclusions	Ref.
2014	Phase II clinical trial	20	Metastatic	HCQ	PFS at 2 months: 10%	Inconsistent autophagy inhibition; negligible clinical effect	[117]
2015	Phase I/II clinical trial	35	Resectable	HCQ + GEM	OS (months): 34.83 (HCQ) vs. 12.27 (controls)	Well tolerated; adverse outcomes unrelated to p53	[118]
2015	Phase I clinical trial	14	Advanced or Metastatic	HCQ + GEM/NP	Median PFS: 6.4 months	Well tolerated	[119]
2017	Phase I clinical trial	9	Unresectable or metastatic	CQ + GEM	Median OS: 7.6 months	Well tolerated; good clinical effect	[120]
2019	Phase I/II clinical trial	119	Advanced or metastatic	HCQ + GEM/NP	OS at 1 year: 41% (HCQ) vs. 49% (controls)	Unimproved 12-month OS	[121]
2020	Phase II clinical trial	64	Potentially resectable tumors	HCQ + GEM/NP	Improved histopathological response; unimproved OS and RFS	Greater tumor pathological response	[122]

*CQ* chloroquine, *GEM* gemcitabine, *HCQ* hydroxychloroquine, *NP* nab-paclitaxel, *OS* overall survival, *PDAC* pancreatic ductal adenocarcinoma, *PFS* progression-free survival, *RFS* recurrence-free survival.

In summary, the clinical effect of autophagy inhibitors on PDAC is often lackluster. The improvement in overall patient response rates may suggest that HCQ or CQ suppresses tumor growth and metabolism in a locally advanced setting [121], while unrestricted invasive tumor behaviors result in a poorer prognosis.

## Future perspectives and concluding remarks

PC is known as the “king of cancers”, and its highly invasive nature makes it is incurable. The dynamic mechanism of activating or inhibiting autophagy in PDAC to overcome invasion may require more research to explore its nature. Thus, we present the following challenges regarding EMT and autophagy in PDAC invasion.

Firstly, p-EMT program is of increasing interest due to its higher invasive and metastatic capacity compared to c-EMT [13, 123, 124]; however, several problems remain to be understood. It is unclear whether PC cells undergoing p-EMT are in an intermediate or terminal state of EMT, and whether the same transcriptional repression mechanisms that drive c-EMT apply to p-EMT [44]. PC cells undergoing p-EMT and c-EMT have differential malignant potentials, and the survival outcomes of PC patients with both EMT procedures in the clinic are unknown.

Secondly, transcriptomics based on EMT-TFs is currently essential for the study of PDAC invasion kinetics [125], but the exact mechanism of EMT-TFs regulating EMT program and how EMT regulates autophagy remains to be studied. (1) How EMT-TFs regulate phenotypic switching (epithelial-mesenchymal interchange) to maintain a high degree of EMT plasticity. The results of several studies have identified different effects of individual EMT-TF and superimposed EMT-TFs on EMT [27, 42, 70], and it is uncertain whether autophagy is involved. (2) Whether *GREM1*, a key regulating factor of PDAC cell heterogeneity [41], regulating autonomous and non-autonomous autophagy has not been elucidated. (3) In the EMT program during PDAC invasion, the expression levels of EMT-TFs are different [41, 42, 126], whether the relationship between EMT-TFs is a programmed cascade or an unplanned crosstalk, and what is the role of autophagy in this. These mechanisms remain the future exploration.

Thirdly, the low transformational ability of stromal cells and their roles in tumor survival has brought significant attention to stroma-targeting-based therapeutics for PC, but studies on the subject have suggested challenges involving the altered stromal environment that predisposes PDAC tissues to invasion: (1) Both tumor-induced increases and decreases in matrix stiffness due to targeting of CAFs promotes invasive behaviors [59, 61]. This paradoxical finding indicates that how to effectively regulate EMT spatial plasticity by altering cell mechanics may be a challenge for the future. (2) Imbalances in the number of stromal cells predispose PDAC to invasive behavior. The optimal ratio of PSCs to stromal cell populations is 0.66–0.83, which leads to optimal growth and invasion of PDAC [58]. In contrast, targeted deletion of CAFs similarly leads to the significant entry of PDAC cells into the EMT program [59]. Whether the reason for these contrasting findings lies in the fact that the EMT program is affected by the activation of the conversion of PSCs to CAFs may also be challenged. Furthermore, non-autonomous autophagy can both promote EMT [35] and provide niches for PDAC invasion. The challenging question then is how an increase in the proportion of non-autonomous autophagy in stromal cells will affect EMT. How will suppression of this ratio affect stromal stiffness and CAFs? Future studies will need to address these possibilities.

Finally, there is a lack of specific drugs targeting autophagy in clinical practice. Systemic autophagy inhibition by HCQ or CQ results in additional toxicity and accelerates organ aging to promote tumor formation [127, 128]. Moreover, the use of autophagy inhibitors in PDAC has been shown to have poor specificity for several reasons: (1) Autonomous autophagy inhibition (pro-EMT) and non-autonomous autophagy inhibition (anti-EMT) have differential heterogeneity in EMT-mediated PDAC invasion, which cannot be selectively inhibited by autophagy inhibitors. (2) Autophagy acts as a survival tool at the interface of multiple cellular processes associated with invasion [129], including growth, metabolism, and immune escape. Inhibition of autophagy may lead to compromised interfaces not related to invasion. (3) Heterozygous deletion of autophagy gene promotes tumor invasion and metastasis, whereas homozygous deletion blocks tumourigenesis rather than invasion [31], suggesting in some sense that the different levels of autophagy inhibition has different effects on PDAC invasion. For these reasons, autophagy inhibitors cannot selectively inhibit specific targets of autophagy, thus making overcoming EMT-mediated PDAC invasion a challenge. Based on the currently accepted understanding of autophagy in PDAC invasion kinetics, specific induction of autonomous autophagic cell death (type II programmed cell death) [130] or inhibition of non-autonomous autophagy may effectively inhibit PDAC invasion (Fig. 6B). Numerous studies have demonstrated the efficacy of autophagic death activation in the treatment of PDAC [131–133]. Furthermore, inhibition of non-autonomous autophagy can reduce tumor-stromal signaling crosstalk and survival-related (proliferation, metabolism, and EMT) niches. This requires drugs that target autophagy to act selectively and specifically on autonomous autophagy-dependent death activation and non-autonomous autophagy inhibition.

In conclusion, EMT and autophagy are targets for the treatment of PDAC, but how to target EMT and autophagy to effectively overcome invasion is the key to successful PDAC therapy. Based on the discussion of cell biology, this review explains that invasion is a survival behavior of PDAC, furthermore, the dynamic plasticity of EMT and the dynamic mechanisms of autophagy endow PDAC with a significant invasive potential. These novel insights may provide ideas for future studies.

## Data Availability

All data generated or analyzed during this study are included in this published article.
